# 

**Published:** 1894-01

**Authors:** 


					﻿THE
Southern Medical Record
A Monthly Journal of Medicine and Surgery.
VOL. XXIV.
Atlanta, Ga., January, 1894.
3^
1.
EDITORS: <
A. W. GRIGGS, M. D.
J. MCFADDEN GASTON, M. D.
WILLIS F. WESTMORELAND, M. D.
LOUIS H. JONES, M. D?
DAN. H. HOWELL, M. D., Bus. Mgr.
Entered at the Postoffice at Atlanta, Ga., as Second-Class Matter.
^MkfaUuaLPrinting Company, Publishers, 27 East Hunter St., Atlanta, Ga,
				

